# Soil respiration and its environmental response varies by day/night and by growing/dormant season in a subalpine forest

**DOI:** 10.1038/srep37864

**Published:** 2016-11-29

**Authors:** Zongda Hu, Shirong Liu, Xingliang Liu, Liyong Fu, Jingxin Wang, Kuan Liu, Xueman Huang, Yuandong Zhang, Fei He

**Affiliations:** 1 College of Resources, Sichuan Agricultural University, 211 Huiming Road, Wenjiang District, Chengdu 611130, Sichuan, China; 2 The Research Institute of Forest Ecology, Environment and Protection, Chinese Academy of Forestry, Dongxiaofu No.2, Haidian District, Beijing 100091, China; 3 Key Laboratory of Forest Ecology and Environment, China’s State Forestry Administration, Dongxiaofu No.2, Haidian District, Beijing 100091, China; 4 Sichuan Academy of Forestry, 18 Xinghui West Road, Chengdu, 610081 Sichuan, China; 5 Research Institute of Forest Resource Information Techniques, Chinese Academy of Forestry, Dongxiaofu No.1, Haidian District, Beijing 100091, China; 6 Division of Forestry and Natural Resources, West Virginia University, P.O. Box 6215, Morgantown, WV, 26506-6125, USA; 7 Dalla Lana School of Public Health, University of Toronto, 155 College Street, Toronto, Ontario M5T 3M7, Canada; 8 Sichuan Engineering Consulting and Research Institute, 201 Yu Sha Road Xinhua Avenue, Chengdu, 610016, Sichuan, China

## Abstract

Comparisons of soil respiration (*R*_*S*_) and its components of heterotrophic (*R*_*H*_) and rhizospheric (*R*_*R*_) respiration during daytime and nighttime, growing (GS) and dormant season (DS), have not being well studied and documented. In this study, we compared *R*_*S*_, *R*_*H*_, *R*_*R*_, and their responses to soil temperature (*T*_5_) and moisture (*θ*_5_) in daytime vs. nighttime and GS vs. DS in a subalpine forest in 2011. In GS, nighttime *R*_*S*_ and *R*_*H*_ rates were 30.5 ± 4.4% (mean ± SE) and 30.2 ± 6.5% lower than in daytime, while in DS, they were 35.5 ± 5.5% and 37.3 ± 8.5% lower, respectively. DS *R*_*S*_ and *R*_*H*_ accounted for 27.3 ± 2.5% and 27.6 ± 2.6% of GS *R*_*S*_ and *R*_*H*_, respectively. The temperature sensitivities (*Q*_10_) of *R*_*S*_ and *R*_*H*_ were higher in nighttime than daytime, and in DS than GS, while they all decreased with increase of *T*_5_. Soil C fluxes were more responsive to *θ*_5_ in nighttime than daytime, and in DS than GS. Our results suggest that the DS and nighttime *R*_*S*_ play an important role in regulating carbon cycle and its response to climate change in alpine forests, and therefore, they should be taken into consideration in order to make accurate predictions of *R*_*S*_ and ecosystem carbon cycle under climate change scenarios.

As an important component of the terrestrial C cycle, soil respiration (*R*_*S*_) in forest ecosystems accounts for 30–90% of the total ecosystem respiration^1^,^2^. Thus, forest *R*_*S*_ and its components, including heterotrophic respiration and autotrophic respiration, have been extensively studied over the past decades^3^,^4^. However, most previous studies on *R*_*S*_, heterotrophic respiration, autotrophic respiration and their responses to environmental changes were conducted during the growing season (GS) and during daytime, and few studies were executed at nighttime and during the dormant season (DS), due mainly to difficulty in field measurement at nighttime and during the DS, in particular, very cold and wet conditions at night time in winter season. Considering greater warming effects (temperature differences) at nighttime than in the daytime, and in winter season than in summer season in Tibetan Plateau^5^,^6^, it is essential to quantify soil CO_2_ fluxes and their temperature responses at nighttime and during the DS.

Soil CO_2_ efflux in the DS plays an important role in the regional, national, and the global carbon balance^7^,^8^, which accounts for 3–50% of annual soil CO_2_ efflux in forests^8^,^9^,^10^. Annual carbon sequestration would be largely overestimated if soil CO_2_ fluxes in the DS were not included^10^. Moreover, several studies by using different approaches including stable isotope methods^11^, modeling methods verified by eddy covariance system^12^, or by using soil respiration monitoring system^13^,^14^ indicated that soil CO_2_ fluxes differed largely between daytime and nighttime. Nevertheless, we have still known little about the proportions and variations of nighttime and the DS *R*_*S*_ in annual soil C fluxes in specific ecosystems.

The temperature sensitivity (*Q*_10_) of *R*_*S*_ is an important fundamental parameter in soil carbon cycle models^15^. It varies with environmental factors and with different components of *R*_*S*_, largely due to the fact that *Q*_10_ is regulated by various biotic and abiotic factors, such as soil temperature and soil water content, microbial biomass, substrate quality, and plant physiological activity^13^,^15^,^16^. However, estimates of *Q*_10_ are mostly based on measurements in daytime and during the GS, and few measurements were conducted in winter and at nighttime. It was reported that *Q*_10_ varied from 1.16 to 24.30 in growing season in sub-alpine forest ecosystem^17^. However, Wang *et al*. found that annual *Q*_10_ ranged from 3.10 to 4.69, indicating that *Q*_10_ varies between the GS and the DS^8^. Additionally, some studies reported that *Q*_10_ value of heterotrophic respiration was lower than that of autotrophic respiration^18^, while other studies showed the opposite result^4^,^13^. Because a slight deviation of *Q*_10_ may cause a huge bias in the estimate of *R*_*S*_^15^, a better understanding of *Q*_10_ of different *R*_*S*_ components in different times can improve our understanding of the roles of forests in regulating carbon cycle and shaping its response to climate change.

The subalpine ecosystems in Tibetan Plateau are considered to be highly sensitive and prone to global warming impacts^19^. The changes in *R*_*S*_ and its temperature sensitivity will have a significant influence on regional and global carbon cycle and their feedback on climate change^20^. Some studies on *R*_*S*_ and heterotrophic respiration have been conducted in subalpine forest ecosystems^9^,^21^, but most of them were based on short term measurements in daytime and during the GS. The CO_2_ fluxes in the DS and at nighttime in the subalpine forests in Eastern Tibetan Plateau have not been fully determined, and the factors controlling the temperature sensitivity of the *R*_*S*_ are even less understood. In the context of global climate change, the rising temperatures may result in greater CO_2_ emissions to the atmosphere from soils in these areas^22^,^23^. Therefore, it is very necessary to accurately estimate *R*_*S*_ and to explore its controlling factors, especially in DS and at nighttime.

In this study, we measured spatial and temporal variations of *R*_*S*_ in a typical oak forest in subalpine mountains and further examined their soil temperature and moisture sensitivity in daytime and at nighttime, and during the GS vs. the DS. We conducted a trenching experiment to partition soil respiration (*R*_*S*_) into rhizospheric (*R*_*R*_) and heterotrophic respiration (*R*_*H*_). The specific objectives of this study were to: (1) quantify the differences of *R*_*S*_*, R*_*H*_, *R*_*R*_ and their responses to environmental factors between daytime and nighttime, and the GS vs. the DS; (2) examine the temperature sensitivity of *R*_*H*_ and *R*_*S*_ and their controlling factors during daytime vs. nighttime, and GS vs. DS.

## Results

### Variations of soil surface CO_2_ fluxes in different time and seasons

**Figure 1:**
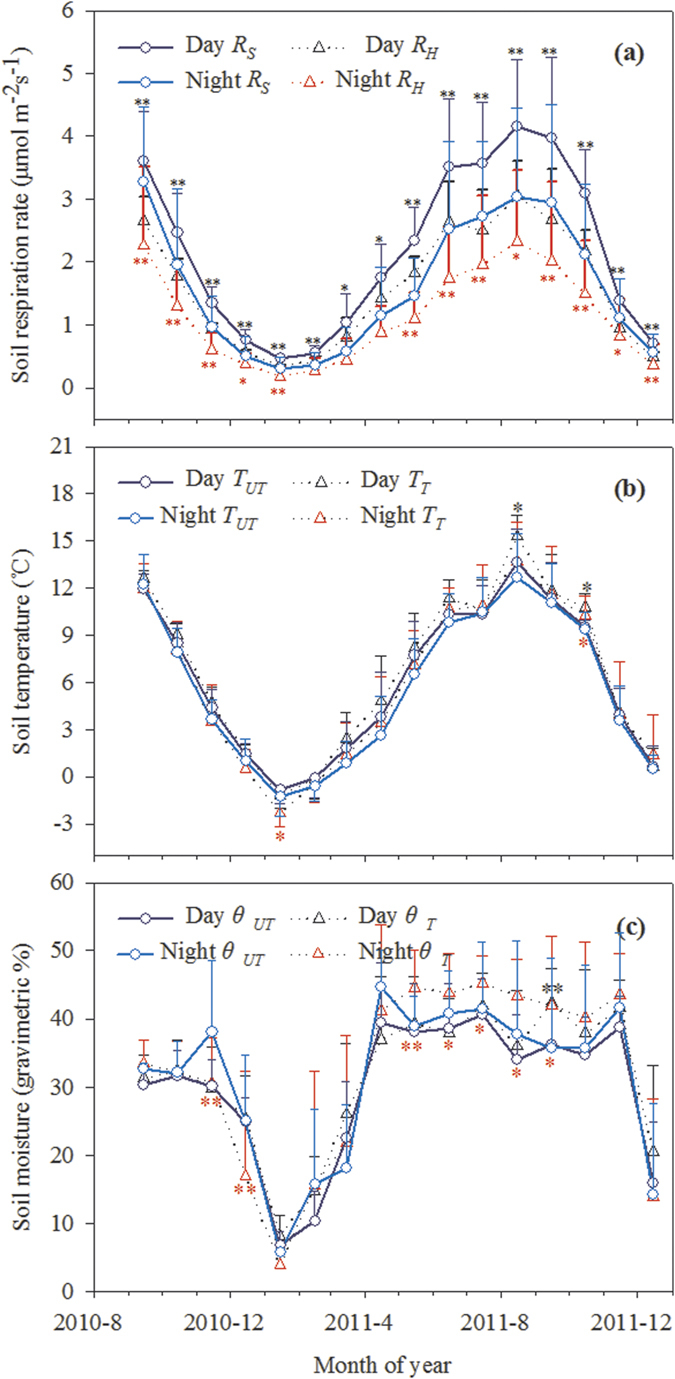
Seasonal dynamics of the mean soil surface CO_2_ fluxes from the daytime (Day *R*_*S*_ and *R*_*H*_) and nighttime (Night *R*_*S*_ and *R*_*H*_), soil temperature (Day *T*_*UT*_ and Night *T*_*T*_) and soil moisture (Day *θ*_*UT*_ and Night *θ*_*T*_) from untrenched and trenched plots in 2010–2011. Vertical bars represent the standard deviation of the mean (n = 9 for untrenched and trenched, respectively). *and **Denote significant difference at *P* = 0.05, *P* = 0.01, respectively. Red and black asterisk represent the significant difference of the day *R*_*S*_ vs. day *R*_*H*_ and night *R*_*S*_ vs. night *R*_*H*_, respectively.

Seasonal dynamics of *R*_*H*_ and *R*_*S*_ showed the similar patterns with that of soil temperature (Fig. 1a and b). The maximum *R*_*S*_ (daytime 4.15 ± 0.20 μmolm^−2^s^−1^; nighttime 3.04 ± 0.13 μmol m^−2^s^−1^) and *R*_*H*_ (daytime 3.05 ± 0.16 μmol m^−2^s^−1^; nighttime 2.33 ± 0.11 μmol m^−2^s^−1^) occurred in August, while the minimum *R*_*S*_(daytime 0.47 ± 0.02 μmol m^−2^s^−1^; nighttime 0.30 ± 0.02 μmol m^−2^s^−1^) and *R*_*H*_ (daytime 0.35 ± 0.02 μmol m^−2^s^−1^; nighttime 0.18 ± 0.01 μmol m^−2^s^−1^) occurred in January. Additionally, soil CO_2_ fluxes was obviously lower in trenched plots than in untrenched plots in the first 6 months after trenching (before December 2010), in order to minimize influence of decomposing roots on *R*_*S*_, we used the data (*R*_*S*_, *R*_*H*_, *T*_5_ and *θ*_5_) from 2011 to analyze the changes in soil surface CO_2_ fluxes.

Both *R*_*S*_ and *R*_*H*_ were significantly higher in daytime than at nighttime (all *P* < 0.05) in all seasons (Table 1). On an average, nighttime *R*_*S*_ accounted for 69.5 ± 4.4% (mean ± std err) and 64.5 ± 5.5% of daytime values in the GS and DS, respectively, while nighttime *R*_*H*_ made up 69.8 ± 6.5% and 62.3 ± 8.5% of the daytime values, respectively. Although *T*_5_ and *θ*_5_ were higher in trenched than in untrenched plots throughout 2011 (Fig. 1), they were not significantly different (*P* > 0.05). Soils generally were drier in DS and wetter in GS (Fig. 1c). Moreover, either *T*_5_ or *θ*_5_ was not significantly different between daytime and nighttime (all *P* > 0.05, Table 2).

The mean *R*_*S*_ rates and *R*_*H*_ rates were significantly higher in the GS than in the DS (*P* < 0.05, Table 1). Overall, DS total mean *R*_*S*_ and *R*_*H*_ (daytime plus nighttime data, respectively, Table 1) account for 27.3 ± 2.5% and 27.6 ± 2.6% of GS total mean *R*_*S*_ and *R*_*H*_, respectively. *θ*_5_ and *T*_5_ were also higher in the GS than in the DS in both daytime and nighttime (Table 2). On average, total mean *T*_*T*5_ (trenched)/*T*_*UT*5_ (untrenched) and *θ*_*T*5_ (trenched)/*θ*_*UT*5_ (untrenched) were 7.4/8.3 and 1.8/1.6 times larger in the GS than in the DS, respectively.

### Factors controlling daytime and nighttime R_S_ and its components

**Figure 2:**
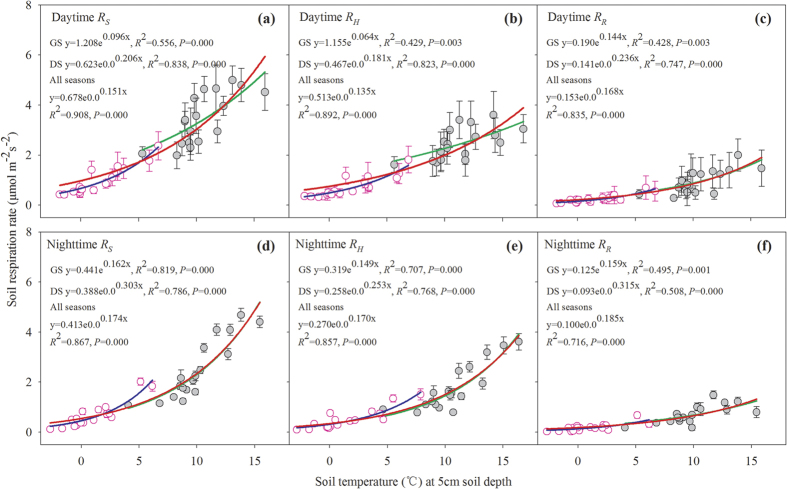
Relationship of soil respiration and its components with soil temperature at 5 cm soil depth in the daytime (**a**–**c**) and nighttime (**d**–**f**). Each value represents the monthly mean of soil CO_2_ flux from the subplot collars measurements for three altitudinal gradients in different seasons (DS pink and GS grey, n = 18. All seasons, n = 36) during 2011. The solid line represents the respiration–temperature relationship estimated according to the model of Eqn (1). All bars indicate mean ± Std Dev (standard deviation). Pink blank circles and grey solid circles represent the monthly mean of soil CO_2_ flux rate in the DS and the GS, respectively.

**Figure 3:**
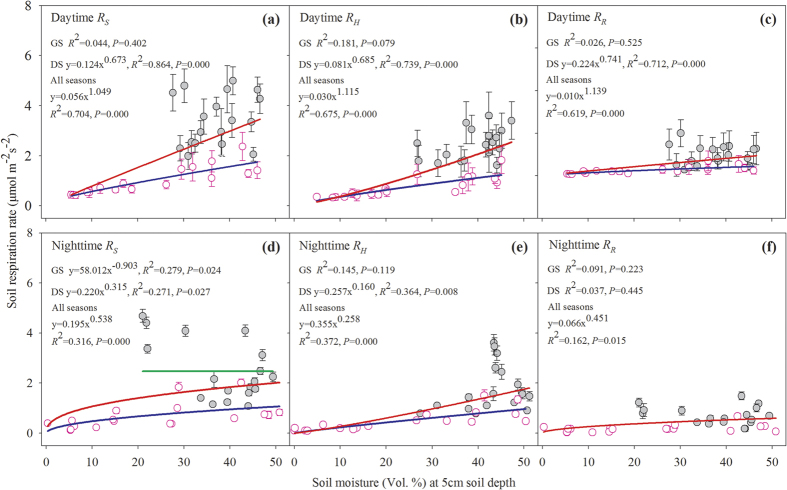
Relationship of soil respiration and its components with soil moisture (volumetric, %) at 5 cm soil depth in the daytime (**a**–**c**) and nighttime (**d**–**f**); Each value represents the monthly mean of soil CO_2_ flux from the subplot collars measurements for three altitudinal gradients in different seasons (DS and GS grey, n = 18. All seasons, n = 36) during 2011. The solid line represents the respiration–moisture relationship estimated according to the model of Eqn (4). All bars indicate mean ± Std Dev (standard deviation). Pink blank circles and grey solid circles represent the monthly mean of soil CO_2_ flux rate in the DS and the GS, respectively.

Both daytime and nighttime *R*_*S*_ and its components followed exponential regression relationships with *T*_5_ (all *P* < 0.001, Fig. 2) and a power model relationship with *θ*_5_ across the seasons (Fig. 3). *T*_5_ alone explained approximately 42.8–90.8% and 49.5–86.7% of the variations in the daytime and nighttime soil C fluxes, respectively. Across all seasons, *θ*_5_ alone explained approximately 61.9–70.4% and 16.2–37.2% of the variations in the daytime and nighttime soil C fluxes, respectively. In DS, the variability in *R*_*S*_ and its components can be explained alone by *θ*_5_ more in daytime than in nighttime. In the GS, we found that only nighttime *R*_*S*_ had significant relationship with *θ*_5_ (Fig. 3d), suggesting that *θ*_5_ was not key factor controlling diurnal dynamics of soil respiration components during the GS.

The combined functions of *T*_5_ and *θ*_5_ (*T*-*θ* model, equation 4) can better explain the variability of *R*_*S*_*, R*_*H*_, and *R*_*R*_, indicating that soil respiration and its components were dominated by the interaction of *T*_5_ and *θ*_5_ rather than a single factor.

### Variations in Q_10_ and its relationships with soil properties

The mean *Q*_10_ values of *R*_*S*_ and *R*_*H*_ were significantly lower in daytime than in nighttime either in the GS or in the DS (all *P* < 0.05, Table 3). Either in daytime or nighttime, we found that *Q*_10_ values of *R*_*S*_ and *R*_*H*_ were significantly higher in the DS than in the GS (all *P* < 0.05).

**Figure 4:**
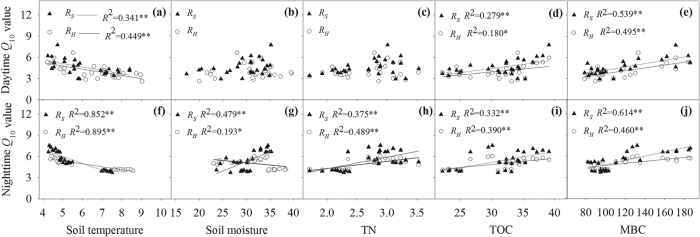
The relationship of *Q*_10_ of *R*_*S*_ and *R*_*H*_ in the daytime (upper panels) and nighttime (lower panels) with soil temperature (**a**,**f**) and soil moisture (**b**,**g**), soil total nitrogen (**c**,**h**), soil total organic carbon (**d**,**i**), soil microbial biomass carbon and nitrogen stocks (**e**,**j**) (n = 27). Each dot represents a *Q*_10_ value that was calculated across seasons for each subplot during the daytime and at nighttime. *and **Denote significant difference at *P* = 0.05, *P* = 0.01, respectively.

*Q*_10_ values of *R*_*S*_ and *R*_*H*_ in daytime and nighttime showed a significantly negative correlation with *T*_5_ (Fig. 4). *θ*_5_ had no significant correlation with *Q*_10_ of either *R*_*S*_ (*P* = 0.077) or *R*_*H*_ (*P* = 0.663) in daytime, while a strong positive/negative correlation was observed between nighttime *Q*_10_ of *R*_*S*_/*R*_*H*_ and *θ*_5_. Either in daytime or nighttime, *Q*_10_ of *R*_*S*_ and *R*_*H*_ had positive correlation with TOC and MBC. TN had no effect on the *Q*_10_ of *R*_*S*_ or *R*_*H*_ in the daytime, but significant effect occurred at nighttime (Fig. 4).

**Figure 5:**
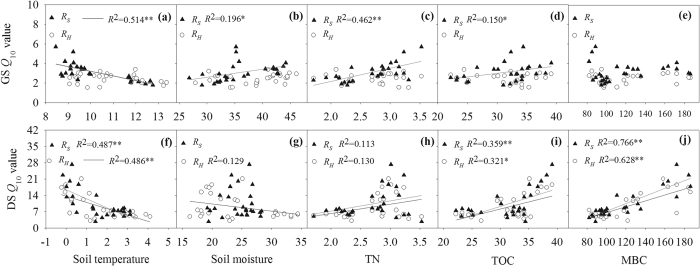
The relationship of *Q*_10_ of *R*_*S*_ and *R*_*H*_ in the GS (upper panels) and the DS (lower panels) with soil temperature (**a**,**f**) and soil moisture (**b**,**g**), soil total nitrogen (**c**,**h**), soil total organic carbon (**d**,**i**), soil microbial biomass carbon and nitrogen stocks (**e**,**j**) (n = 27). Each dot represents a *Q*_10_ value that was calculated across daytime and nighttime observations for each subplot in the GS and the DS. *and **Denote significant difference at *P* = 0.05, *P* = 0.01, respectively.

*Q*_10_ of *R*_*S*_in the GS and the DS had significant correlations with *T*_5_ (Fig. 5). *Q*_10_ of *R*_*H*_ had negative correlations with *T*_5_ in the DS but not in the GS. In contrast, *θ*_5_ showed significant relationship with *Q*_10_ of *R*_*S*_ in the GS, but not in the DS. Additionally, in the GS, *Q*_10_ of *R*_*S*_ had positive correlation with TOC and TN, but *Q*_10_ of *R*_*H*_ was not affected by TOC and TN. In the DS, however, *Q*_10_ of both *R*_*S*_and *R*_*H*_ was positively influenced by TOC and MBC (Fig. 5).

## Discussion

### Comparisons of soil respirations in daytime vs. nighttime and the GS vs. the DS

In this study, we found that the measured *R*_*S*_ and *R*_*H*_ was 30.5 ± 4.4% (mean ± SE) and 30.2 ± 6.5% lower at nighttime than in daytime during the GS, while they were 35.5 ± 5.5% and 37.3 ± 8.5% lower during the DS, respectively. The changes in soil C fluxes in daytime and at nighttime were due mainly to variations of soil temperature and moisture. Additionally, previous studies reported that the diurnal variability of soil CO_2_ efflux was affected by the turnover of recent photosynthate^11^, microbial growth^24^, plant biological activities^25^, and allocation of photosynthetic C^11^,^26^. In the daytime, favorable soil temperature and light condition can promote the enhancement of microbial metabolism. Moreover, higher daytime *R*_*S*_ rates may result from greater translocation of high photosynthesis from the plant shoots to the roots during the daytime relative to at nighttime. The differences in the content and quality of SOC were also reported to determine high and low *R*_*S*_ in previous studies^27^,^28^,^29^.

Previous studies shows that *R*_*H*_ for a certain time after trenching treatment may be increased in trenched plots^30^, or kept almost unchanged, or decreased^31^. In this study, we observed that *R*_*H*_ rates in trenched plots significantly lower than in untrenched plots for over 2 months after trenching (September 2010; Fig. 1). The results are consistent with that previously reported in different forest types^21^,^32^. In our study, we found that *R*_*H*_ was the dominant component of *R*_*S*_ during the DS and the GS in 2011. The *R*_*H*_ accounted for 72.2 ± 1.2% (mean ± SE) of the whole year *R*_*S*_, with 73.9 ± 2.0% of *R*_*S*_ in the DS and 71.7 ± 1.3% of *R*_*S*_ in the GS, respectively (Table 1). Our findings are within the range of 16–80% of the contribution of *R*_*H*_ to *R*_*S*_ reported previously in temperate coniferous forests^7^,^33^. However, there were no significant differences in proportion of *R*_*H*_ to *R*_*S*_between DS and GS. Higher rates of *R*_*H*_ in DS were likely due to relatively high metabolic reaction of roots and soil microbial activity for maintaining respirations in the study stands in the winter. Our results suggest that *R*_*H*_ was the dominant component of *R*_*S*_, indicating a dominant control of microorganism-associated respiration on *R*_S_ in the subalpine forest. In the DS, we supposed that soil microbial activity is still functioning to generate CO_2_ fluxes by decomposing soil organic matters, while the root activity of plants is inhibited in winter because plants stop growing in cold temperature^9^, and consequently, ending up with a high proportion of *R*_*H*_ to total *R*_*S*_ (Table 1). Therefore, soil C fluxes in the DS must be taken into consideration when assessing the carbon sink/source patterns of the subalpine forests.

### Environmental factors influencing soil CO_2_ flux

In this study, the daytime and nighttime *R*_*S*_ and its components were significantly influenced by the interactions of *T*_5_ and *θ*_5_. Our results are similar to the findings observed in other forests^32^,^34^. In this study, however, a new finding is that *T*-*θ* model fits the observations better in the DS than the GS for the all components of *R*_*S*_, indicating that *R*_*S*_ and its components can be better predicted through *T*_5_ and *θ*_5_ in the DS than in the GS. This provides a simple but effective basis for estimating soil respirations in winter.

We also found that *θ*_5_ alone didn’t impact either the daytime or the nighttime *R*_*H*_ and *R*_*R*_ in the GS but *T*_5_ did so, suggesting that *T*_5_ rather than *θ*_5_ is a main factor controlling *R*_*H*_ and *R*_*R*_ in the GS. Daytime *R*_*S*_ had no relationship with *θ*_5_, but nighttime *R*_*S*_ decreased linearly with increasing *θ*_5_ in the GS (Fig. 3d). This is because a high precipitation frequency at nighttime in the GS decreases *T*_5_ and consequently suppresses soil CO_2_ efflux at nighttime. The different relationships of *R*_*S*_ and its components with *θ*_5_ between the GS and the DS (Fig. 3) suggest that the different roles of *T*_5_ and *θ*_5_ as independent environmental variables in GS vs. the DS should be specifically taken into consideration when we predict soil respirations.

### Constraints on Q_10_

There have been inconsistent conclusions on *Q*_10_ values of *R*_*S*_ and its components in previous studies^35^. Moreover, few studies have compared *Q*_10_ of *R*_*S*_ and its components in the daytime vs. the nighttime and the GS vs. the DS, especially at the high altitude in subalpine regions. In this study, the *R*_*S*_ and *R*_*H*_ at nighttime and during the DS are apparently more sensitive to temperature than that during daytime and the GS (Table 3). Our result is in line with some of previous studies that *Q*_10_ values were higher at lower temperature^17^,^36^, but conflicts with other reports that *Q*_10_ was not characterized by a reflection of temperature change^37^. The high temperature sensitivity of *R*_*S*_ and *R*_*H*_ at low temperature environments may be due to a high rate of substrate utilization and changes in microbial populations and microbial activity aiming to maintain high decomposition rates^37^,^38^. This indicates that *R*_*S*_ or *R*_*H*_ at nighttime and during the DS might be more sensitive to temperature change than during daytime and the GS. In the GS, other parameters, such as substrate supply by roots and leaf area index in relation to phenology, may be more important than temperature in controlling the carbon flux rates^14^,^39^.

In this study, soil moisture did not influence *Q*_10_ values of *R*_*S*_ and *R*_*H*_ in the daytime and the DS, but we found that *Q*_10_ values of *R*_*S*_ increased with increasing *θ*_5_ at nighttime and the GS, while *Q*_10_ of *R*_*H*_ decreased with increasing *θ*_5_ at nighttime (Figs 4 and 5). The similar results were also reported in the sub-alpine forests of the Eastern Qinghai-Tibet Plateau^17^. Soil moisture may cause changes in microbial community structure and soil mineralization rate^40^,^41^, and furthermore affect sensitivities of their biotic and physicochemical processes to temperature^42^. In addition, plant-microbe-soil interactions and plant metabolism could contribute to *Q*_10_ values of *R*_*S*_ and its components^43^.

The availability of soil substrates can influence microbial metabolism^44^. Therefore, increased availability of either soil carbon or nitrogen is expected to stimulate microbial growth and activity in soils^45^,^46^, leading to the subsequent changes in *Q*_10_ of *R*_*S*_ and *R*_*H*_^47^. In this study, we found that TOC and MBC were key factors affecting the temperature sensitivities of both daytime *R*_*S*_ and *R*_*H*_ and nighttime *R*_*S*_ and *R*_*H*_ during DS (Figs 4 and 5). Therefore, in addition to soil temperature and moisture, the changes in TOC and MBC additionally explain the temperature sensitivity of soil C flux^47^. Our results suggest that if a constant seasonal *Q*_10_ is used in the models for estimating soil CO_2_ efflux, we cannot make accurate prediction on future soil CO_2_ losses^15^.

### Uncertainty of trenched effects on soil respirations

**Figure 6:**
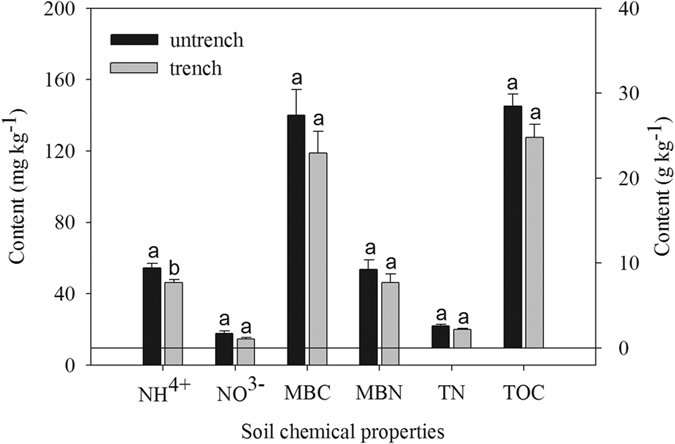
Mean contents of inorganic nitrogen (IN, NH^4+^+NO^3−^), soil microbial biomass carbon (MBC) and nitrogen (MBN), total nitrogen (TN), soil total organic carbon (TOC) in the soil of trenched and untrenched plots (n = 27), respectively. Bars with the different lower-case letters indicate mean ± SE (standard error) within trenched and untrenched subplots that are significantly different (*P* *<* 0.05).

The trenching method for root exclusion is generally used to estimate *R*_*H*_, especially in forest ecosystems although some uncertainties exist on its accuracy and interpretation of the results^48^,^49^. One possible uncertainty may originate from the change in soil environmental conditions caused by trenching^50^. In this study, we found a higher level of *T*_5_ and *θ*_5_ through 2011 (daytime: 9.0% and 7.6%, nighttime: 8.6% and 7.7%, respectively, absolute difference) in the trenched plots compared with the untrenched plots, which may lead to an overestimation of *R*_*H*_^51^. However, we did not find any significant trenching effect on *T*_5_ or *θ*_5_ in either the GS or the DS (Table 2). Therefore, the trench-induced changes in soil microclimate can be neglected in this study. Another possible impact of trenching on soil chemical parameters is that trenching can change the nutrient conditions and thus alter microbial activities^52^. In the present study, the soil carbon and nitrogen components were different degrees higher in untrenched plots than in trenched plots (Fig. 6), and suggested that the difference could be attributed to the suppression of photosynthetic products supply to the rhizosphere. Additionally, we used a linear regression model to determine this estimation error^4^. Our results showed that the trenched respiration contribution decreased by 4.3% and 3.9% after this correction in daytime and nighttime. We believe this process is unlikely to affect our estimation of *R*_*H*_ due to its minor contribution (5–8%) to soil respirations in temperate forests^4^,^18^,^53^. Although some under- or over-estimation may be inevitable, we feel that trenching method is reasonable in this study.

Previous studies suggested that the fine roots may quickly decompose after trenching and cause a high CO_2_ fluxes^4^,^52^. In this study, we found that the following third month after trenching, the *R*_*S*_ was obviously higher in untrenched plots than in trenched plots (Fig. 1, *P* = 0.000). The similar phenomenon were also reported by Wang and Yang^32^ in the temperate deciduous and evergreen pine forest^32^. In fact, we used the measurement data after 6 months since the onset of trenching experiment in order to avoid the impact of rapid CO_2_ flush caused by trenching. Previous studies indicated that priming effects from dead roots after trenching can be avoided if it is properly managed^4^,^54^. The trenching had no significant influence on *T*_5_ and *θ*_5_ in this study, whereas controlling mechanisms on the temporal and spatial variation of *R*_*H*_ were more complex. Advanced techniques (e.g. stable ^14^C and ^13^C isotope tracing) and laboratory incubation experiments based on plant physiology are needed to elucidate the variation mechanism of *R*_*H*_ and rhizospheric respirations. Moreover, we observed in the study site that most roots (<5 mm) of *Q. aquifolioides* forest were distributed at the soil depth of 0–30 cm and few roots existed below the depth of 50 cm. Trenching down to the soil depth of 70 cm in this study should be deep enough to exclude the root impacts, and our results justified it to some extent.

## Conclusion

In subalpine forests, *R*_*S*_ and its components are obviously greater in the GS than in the DS and are higher during daytime than at nighttime. The temporal variations of daytime and nighttime *R*_*S*_ and its components can be well explained by the interactions of soil temperature and moisture regardless of seasons. *R*_*S*_ and its components are more sensitive to temperature in the DS than in the GS, and at nighttime than in daytime. Additionally, the substrate availabilities affected differently on *Q*_10_ of *R*_*S*_ and *R*_*H*_ on a daily and seasonal scale. Our findings suggest that soil CO_2_ emission throughout the DS and at nighttime in the high-altitude subalpine area plays a vital role in annual soil carbon budgets and their responses to climate change. Temporal variations of *R*_*S*_ and its responses to environmental factors need to be considered in order to accurately predict changes in soil CO_2_ fluxes.

## Materials and Methods

### Site description

The research was conducted in Wolong Nature Reserve in Sichuan Province, China (102°58′–103°06′ E, 30°53′–58′ N), which is located at the Balang Mountain in the east branch of Qionglai Mountains, southeast of Tibetan Plateau. The brown mountainous soil (Chinese classification) is the main soil type across the whole study area of the *Quercus aquifolioides* distribution. The area is characterized by the typical Tibetan Plateau climate with distinct wet and dry seasons. At the 2700 m a.s.l., annual mean air temperature is about 8.4 °C, with the average minimum/maximum (January) and maximum/minimum (July) air temperature being −1.7/+5.1 and +17/6.3 °C, respectively. The annual precipitation is 861.8 mm, of which 68.1% occurs from May to September. According to the field observation, the average soil temperature at 5 cm soil depth in 2011 was 5.2 ± 4.2 °C (mean ± std. deviation) at 3549 m a.s.l., 4.8 ± 5.3 °C at 3091 m a.s.l., and 7.4 ± 5.3 °C at 2551 m a.s.l., with corresponding soil moisture values of 29.1 ± 10.3%, 30.4 ± 15.8% and 34.6 ± 11.1%, respectively. In the study site, *Q. aquifolioides* is the dominant species of this typical oak forest, while other species include *Cotoneaster horizontalis, Daphne tangutica, Deyeuxia levipes, Oryzopsis munroi, Pedicularis davidii, Athyrium pachyphlebium*.

### Experiment design

Three *Q. aquifolioides* forest experimental sites were set up along the altitude gradient within its optimal spatial coverage (2551, 3091 and 3549 m, Table 4), on the southeast slope of Balang Mountain. At each site, three 20 m × 20 m replicate plots were established with a distance of 10 m between plots. In each plot, three 80 cm × 80 cm subplots were randomly deployed for trenching experiment and a buffer area was set with 10 m apart from each other. On 18 June 2010, at the outside edges of each subplot, a trench of 0.7 m deep (to the bedrock or below where few roots existed) was dug using a steel knife and shovel. On this study site, we observed that most roots (diameter <5 mm) were distributed in soil between 0 to 30 cm in depth in *Q. aquifolioides* forest, and few roots existed below the depth of 50 cm. To prevent the trenched plots from root encroachment, we lined the trenches with double-layer plastic sheets, and then refilled them carefully with the same soil. At each trenched subplot, one PVC (20 cm inside diameter × 8 cm in height) collar was installed into soil to a depth of 5 cm for CO_2_ efflux sampling (*R*_*H*_). Furthermore, all aboveground vegetation was carefully removed with minimal soil disturbance to keep a free of seedlings and herbaceous vegetation in these subplots throughout the course of experiment (from Sept, 2010 to Dec, 2011). Another three PVC collars were randomly inserted into the soil to a depth of 5 cm within 3 m around each trenched subplot (including the litter layer) to measure the *R*_*S*_, which was considered as the total soil respirations rate. Once inserted, the collars were left unmoved during the entire study period. Our first measurements of *R*_*S*_ started two and half months later after the onset collar installation (mid-September 2010). Site characteristics are listed in Table 4 and Fig. 6.

### Soil respirations, temperature and moisture measurements

In order to minimize the possible influence of live and dead roots in trenched plots decay, soil CO_2_ fluxes was measured 75 days later since the onset of trenching experiment^32^. In this study, we actually used the measurement data 6 months later since the onset of trenching for minimizing the effects of root decomposition in trenched plots. Therefore, soil CO_2_ flux in trenched plots can be regarded as the trench respiration (*R*_*H*_), while *R*_*R*_ was the difference between *R*_*S*_ and *R*_*H*_^18^,^21^. Soil respirations rates in the untrenched plots (*R*_*S*_) and the trenched (*R*_*H*_) were measured monthly from September 2010 to December 2011 (8th~18th per month except rainy days). At the time of *R*_*H*_ and *R*_*S*_ measurements, Soil temperature (*T*_5_) and moisture (volumetric water content, *θ*_5_) at 5 cm depth were measured automatically using the soil temperature probe and an attached Theta probe ML2x (Delta-T Devices, Cambridge, England) equipped with a Li-8100A soil CO_2_ flux system (LI-Cor Inc., Lincoln, NE, USA). In the daytime, respiration was measured between 9:00 and 16:00 hours (local time), while at nighttime it was measured between 20:00 and 4:00 hours^55^,^56^. The *R*_*S*_ and *R*_*H*_ were measured continuously in two cycles at each collar, 3 minutes per each cycle. The two measurements were averaged to produce the collar’s mean *R*_*S*_ and *R*_*H*_ rates and mean *T*_5_ or *θ*_5_. The average values of the 9 (3 subplots × 3 replicates) measurements at each altitude for *R*_*H*_, *T*_*T*_ and *θ*_*T*_ (trenched), respectively, while the three collars’ measurements from the nearby trenched subplots were averaged to produce one mean value of *R*_*S*_, *T*_*UT*_ and *θ*_*UT*_ (untrenched), respectively. The average values of the 27 (3 subplots × 3 replicates × 3 altitudes) measurements around each trenched subplot in each month for soil CO_2_ rates, *T*_*5*_and *θ*_*5*_ were used for data analysis.

### Soil chemical property measurements

Soil was sampled at 0–30 cm depth with a sample ring kit with closed ring holder (Eijkelkamp 07.53.SC, Holland) in mid-April, mid-August and mid-November of 2011, respectively. In each subplot, near each PVC collar (within 80 cm) three soil cores were collected and mixed thoroughly into one sample. Altogether, nine samples from each plot were stored in a cooler before being carried back to the lab. Roots, gravel, and other miscellany of things in the samples were manually removed and the soil was sieved with a 2 mm sieve. Each sample was divided into two parts. One was stored at room temperature and was air-dried for analysis of soil chemical properties, the other was immediately deposited in a refrigerator at 4 °C for soil microbial biomass measurement that was conducted within 3–4 days.

The contents of soil NH^4+^ and NO^3−^ were determined by using the indophenol blue colorimetric method and dual-wavelength spectrophotometry, respectively. Soil TOC was determined with the potassium dichromate oxidation heating method, and TN was determined with the semimicro Kjeldahl method using the air-dried soil. The measurements of soil microbial biomass carbon (MBC) and nitrogen (MBN) was performed by using the chloroform fumigation extraction method according to Vance *et al*.^57^.

### Calculation of temperature sensitivity

Based on the measured data in 2011, an exponential equation (Van’t Hoff model, T-model; Eq. 1) was formulated to interpret the relationship between *R*_*S*_, *R*_*H*_ or *R*_*R*_ and soil temperature (*T*_5_) at 5 cm soil depth in three altitudinal plots. The temperature response of *R*_*S*_, *R*_*H*_ or *R*_*R*_ was estimated in this study according to the following equations Eqs 1 and 2, respectively.


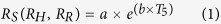



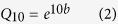


Where *R*_*S*_, *R*_*H*_ are the mean soil surface CO_2_ fluxes (μmolm^−2^s^−1^), *a* and *b* are fitted parameters. The *b* values were used to calculate the temperature sensitivity (*Q*_10_). We calculated daytime and nighttime respiration *Q*_10_ across seasons for each subplot. Additionally, we calculated the DS (early November through late April) and the GS (early May through late October, the growing season was determined by the phenological events of dominant *Q. aquifolioides) Q*_10_ values across daytime and nighttime measurements.

### Relationship between R_S_ (R_H_ or R_R_) and soil moisture

A power equation (*θ*-model; Eq. 3) was used to describe the relationship between *R*_*S*_, *R*_*H*_ or *R*_*R*_ and soil moisture (*θ*_5_) at 5 cm soil depth at three altitudinal plots. A logarithm transformation was performed on *R*_*S*_ and *R*_*H*_ to achieve linearity and homogeneity of variance test, respectively, and was used as a function of *T*_5_ and *θ*_5_^4^ (T-*θ* model; Eq. 4).


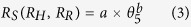



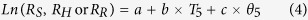


Where *a, b* and *c* are the fitted parameters.

### Statistical analyses

A repeated measures analysis of variance (ANOVA) was used to test for differences of treatment (trenched and untrenched) on soil CO_2_ rates, *T*_5_ and *θ*_5_ based on the measurement data. A one-way ANOVA was used to compare the differences of soil CO_2_ flux, *T*_5_ and *θ*_5_ between daytime and nighttime in different seasons. Based on the monthly mean of soil CO_2_ fluxes at each altitudinal gradient, the dependence of *R*_*S*_, *R*_*H*_, and *R*_*R*_ on *T*_5_ or *θ*_5_ was investigated using nonlinear and multiple linear regression models. A linear regression was used to analyze the relationship of *Q*_10_ with *T*_5_, *θ*_5_, and other soil properties in daytime/nighttime and seasons across 27 subplots (3 subplots × 3 replicates × 3 altitudes). The significant differences of *Q*_10_ values between daytime and nighttime, between the DS and the GS, were tested using the ANOVA at α = 0.05, respectively. In order to ensure the comparability of *R*_*H*_ and eliminate the effects of trenching treatment on *R*_*H*_, measured data were corrected where *T*_5_ and *θ*_5_ diverged on trenched and untrenched plots throughout the measurements in 2011. We used a multiple linear regression function to evaluate the trenching effect on soil C fluxes. All statistical analyses were performed using SPSS (version 19.0 for Windows).

## Additional Information

**How to cite this article**: Hu, Z. *et al*. Soil respiration and its environmental response varies by day/night and by growing/dormant season in a subalpine forest. *Sci. Rep.* **6**, 37864; doi: 10.1038/srep37864 (2016).

**Publisher's note:** Springer Nature remains neutral with regard to jurisdictional claims in published maps and institutional affiliations.

## Figures and Tables

**Table 1:** The differences of *R*_*H*_ and *R*_*S*_ (μmol m^−2^s^−1^) between daytime and nighttime in the growing season and dormant seasons.

Components	Diel	GS	DS	All seasons
*R*_*S*_	Daytime	3.44 (0.09)aA	0.98 (0.05))aB	2.21 (0.09)a
	Nighttime	2.47 (0.05)bA	0.67 (0.02)bB	1.57 (0.04)b
*R*_*H*_	Daytime	2.47 (0.07)aA	0.73 (0.04)aB	1.60 (0.06)a
	Nighttime	1.77 (0.04)bA	0.49 (0.02)bB	1.13 (0.03)b

The different lowercase letters in a column represent significant difference between daytime and nighttime. The different uppercase letters represent significant difference between the GS and the DS at *P* = 0.05 level. Values in parentheses are standard errors of means for N = 162.

**Table 2:** The statistics of soil temperature and moisture between daytime and nighttime in the trenched and untrenched plots in different seasons of the *Q. aquifolioides* forest during 2011.

Plots	*T*_5_/*θ*_5_	Diel	GS	DS	All seasons
Untrenched	*T*_5_	Daytime	10.46 (0.20)aA	1.53 (0.21)aB	5.99 (0.29)a
		Nighttime	9.97 (0.12) aA	0.93 (0.10)bB	5.94 (0.72)a
Trenched		Daytime	11.33 (0.20)aA	1.74 (0.21)aB	6.53 (0.29)a
		Nighttime	10.66 (0.12)bA	1.22 (0.10)aB	5.45 (0.72)a
Untrenched	*θ*_5_	Daytime	37.04 (1.23)aA	22.26 (2.60)aB	29.65 (1.67)a
		Nighttime	38.35 (0.41)aA	23.32 (0.75)aB	30.84 (0.49)a
Trenched		Daytime	39.19 (0.63)aA	24.62 (1.21)aB	31.91 (0.83)a
		Nighttime	43.14 (0.29)bA	23.29 (0.80)aB	33.21 (0.53)a

The different lowercase letters in a column represent significant difference between daytime and nighttime in the GS, the DS and all seasons, respectively. The different uppercase letters represent significant difference between the GS vs. the DS in the daytime or at nighttime at *P* = 0.05 level. Values in parentheses are standard errors of means for N = 162.

**Table 3:** Mean *Q*_10_ in the daytime and at nighttime during the GS and the DS.

Daily	GS		DS	
	*R*_*S*_	*R*_*H*_	*R*_*S*_	*R*_*H*_
Daytime	2.84 (0.26)aA	2.20 (0.13)aA	7.42 (0.83)aB	6.41 (0.61)aB
Nighttime	3.94 (0.28)bA	2.72 (0.08)bA	20.02 (3.92)bB	18.99 (3.48)bB

The different lowercase letters in a column represent significant difference between the daytime and nighttime in the GS and the DS, respectively. The different uppercase letters represent significant difference between the GS vs. the DS at *P* = 0.05 level. Values in parentheses are standard errors of means for N = 27.

**Table 4:** Site characteristics of the three sites in *Quercus aquifolioides* forest.

Altitude (m)	Density (clumps ha^−1^)	TD (cm)	HT (m)	CR (m^2^)	Litter layer (cm)	pH	BD (g/cm^3^)
3549	3897	3.62 (1.44)a	2.45 (0.82)a	4.47 (3.20)a	1.46 (1.11)a	6.75 (0.75)a	1.46 (0.59)a
3091	2167	4.56 (1.71)b	4.50 (0.90)b	8.69 (4.45)b	2.24 (1.01)b	5.34 (0.09)b	1.24 (0.04)a
2551	2142	6.74 (3.09)c	6.43 (1.48)c	10.99 (7.84)b	2.13 (1.08)b	5.20 (0.21)b	1.47 (0.05)a

TD, HT, CR and BD refer to stem diameter at 50 cm from ground, height, crown of the *Q. aquifolioides* and bulk density, respectively. Values in parentheses are standard deviations of means.
